# Immunity and inflammation: the neglected key players in congenital heart disease?

**DOI:** 10.1007/s10741-021-10187-6

**Published:** 2021-12-02

**Authors:** Laura M. Wienecke, Sarah Cohen, Johann Bauersachs, Alexandre Mebazaa, Benjamin G. Chousterman

**Affiliations:** 1 Department of Cardiology and Angiology, Hannover Medical School, Carl-Neuberg-Str. 1, 30621 Hannover, Germany; 2 Department of Anaesthesiology and Critical Care, Lariboisière University Hospital, DMU Parabol, AP-HP Paris, France; 3 Inserm U942 MASCOT, Université de Paris, Paris, France; 4 Congenital Heart Diseases Department, M3C Hospital Marie Lannelongue, Université Paris-Saclay, Plessis-Robinson, Paris, France; 5 Department of Cardiology, Angiology and Respiratory Medicine, Heidelberg University Hospital, Heidelberg, Germany

**Keywords:** Congenital heart disease, Inflammation, Heart failure, Thymectomy, COVID-19

## Abstract

Although more than 90% of children born with congenital heart disease (CHD) survive into adulthood, patients face significantly higher and premature morbidity and mortality. Heart failure as well as non-cardiac comorbidities represent a striking and life-limiting problem with need for new treatment options. Systemic chronic inflammation and immune activation have been identified as crucial drivers of disease causes and progression in various cardiovascular disorders and are promising therapeutic targets. Accumulating evidence indicates an inflammatory state and immune alterations in children and adults with CHD. In this review, we highlight the implications of chronic inflammation, immunity, and immune senescence in CHD. In this context, we summarize the impact of infant open-heart surgery with subsequent thymectomy on the immune system later in life and discuss the potential role of comorbidities and underlying genetic alterations. How an altered immunity and chronic inflammation in CHD influence patient outcomes facing SARS-CoV-2 infection is unclear, but requires special attention, as CHD could represent a population particularly at risk during the COVID-19 pandemic. Concluding remarks address possible clinical implications of immune changes in CHD and consider future immunomodulatory therapies.

## Introduction

Advances in medical care have led to a substantially higher population of patients with congenital heart disease (CHD) with an estimated prevalence of around 1.9 million children and 2.3 million adults with CHD in Europe [1]. However, these patients still suffer from markedly higher and premature morbidity and mortality [2, 3]. Heart failure (HF) constitutes the common central issue in CHD, despite the wide heterogeneity of various congenital heart defects [2]. Indeed, leading causes of death in adults with CHD (ACHD) are heart failure (43%), infections such as pneumonia and endocarditis (12%), as well as sudden cardiac death (7%) [2].

CHD-related HF (CHD-HF) should always be considered separately from non-congenital HF as they arise from different etiologies. CHD-HF is more often characterized by a leading right rather than left ventricular failure and includes a lifelong chronic cardiac impairment with frequent surgical corrections or interventions (depending on the distinct malformations). Clinical care of CHD patients focuses predominantly on hemodynamics and treatment of complications and comorbidities [4]. To date, prevention as well as evidence-based treatments for CHD-HF are lacking apart from open heart surgery or interventional therapy.

Systemic chronic inflammation and immune activation have been identified as crucial drivers of disease development and progression, and they came up as promising therapeutic targets in non-congenital HF and other cardiovascular diseases [5]. However, they have not been evaluated sufficiently for the management of CHD, even though recent reports describe several elevated inflammation-related markers with prognostic impact in CHD [6, 7].

The immune system includes innate and adaptive immune strategies to combat pathogen- or tissue damage-associated harm of the host organism. Next to innate and adaptive immune activation caused by HF itself, thymus removal could have an impact on adaptive immunity in CHD [8]. Corrective cardiothoracic surgery of malformations using a median sternotomy routinely includes the removal of thymus tissue to access the operation field—the heart and its great vessels. Surgical repair in CHD is often performed during the first years of life, which represents a crucial period regarding thymic lymphocyte production. As a consequence, several studies observed immune alterations such as pronounced immune senescence with reduced naïve and increased senescent T cell subsets [8]. Therefore, CHD patients represent a very interesting and complex cohort from an immunological point of view.

CHD may originate from genetic or idiopathic causes and displays a large phenotypic diversity that may explain the variety of observed cardiac and immune alterations. The most common genetic syndromes linked to congenital heart malformations are Down syndrome (Trisomy 21), DiGeorge syndrome, and Noonan syndrome [9]. All three are related to immune deficiency, senescence, and increased risk for malignancies of the hematopoietic system, suggesting an underlying link between the development of the heart and of the immune system.

In this review, we aim to highlight the current knowledge and clinical implications regarding innate and adaptive immune alterations, inflammation and thymectomy in children and adults with CHD. Especially considering the COVID-19 pandemic, we will shed light onto the potential impact of T cell senescence, genetic syndromes, and chronic inflammation on patient outcomes upon SARS-CoV-2 infection. Although immunologists have investigated the immune changes in CHD for decades, a lot of cardiologists, pediatric cardiologists, and cardiothoracic surgeons are not aware of these insights.

## Causes and consequences of inflammation and immune changes in CHD

**Fig. 1:**
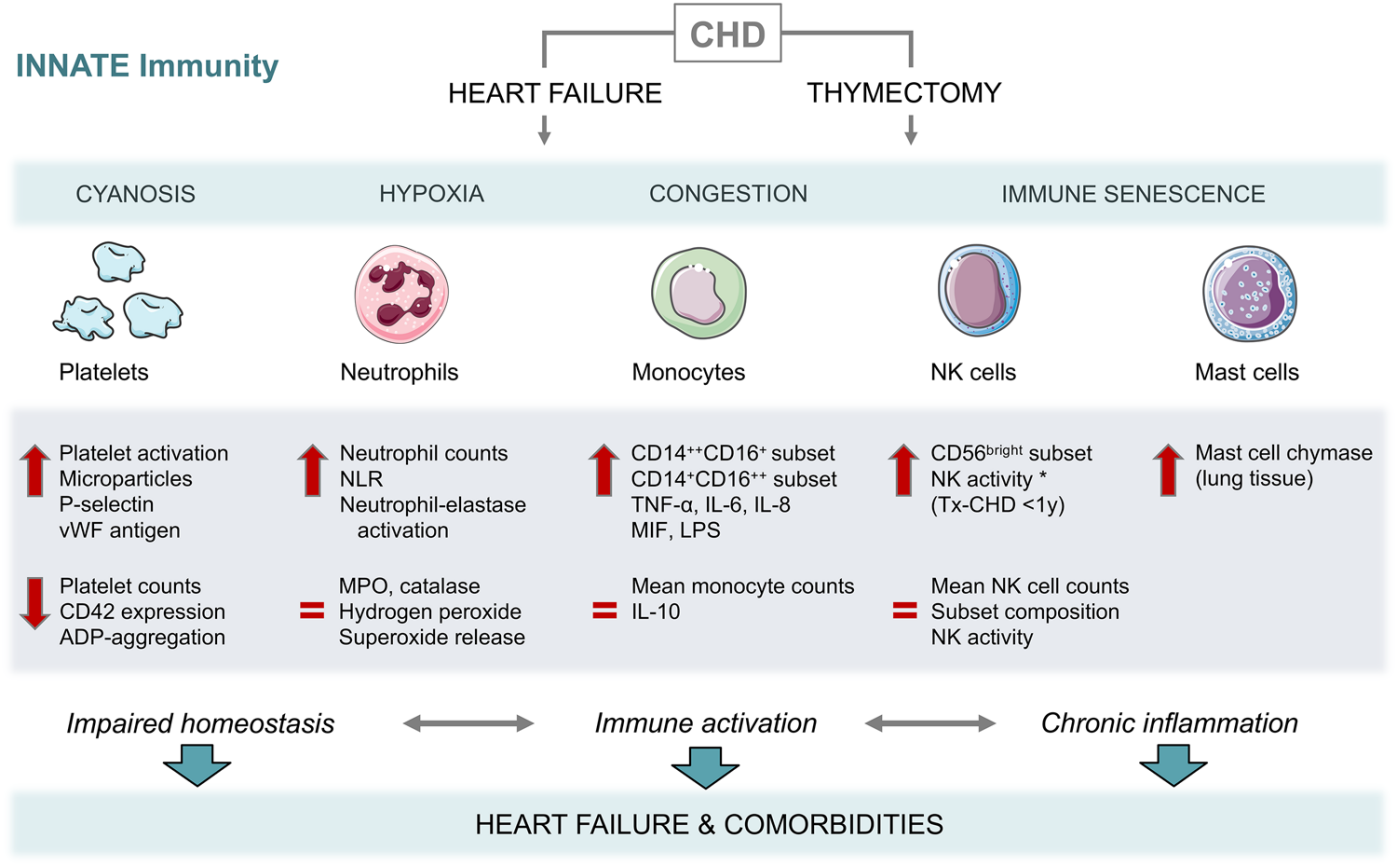
**Innate immune mediators. **Congenital heart disease (CHD)-associated heart failure and thymectomy are related to alterations of innate immune effectors contributing to impaired homeostasis, immune activation, and chronic inflammation. These changes can impair heart failure and promote comorbidities. Bold red flashes indicate increased or decreased parameters; equal sign indicates unchanged parameters in CHD compared to healthy controls. *Indicates increased NK cell activity only in CHD patients who had been thymectomized at the age of 1 year or younger. Servier Medical Art PowerPoint templates were used for graphical illustration. *ADP* adenosine diphosphate, *CD* cluster of differentiation, *CHD* congenital heart disease, *IL* interleukin, *LPS* lipopolysaccharide, *MIF* macrophage migration inhibitory factor, *MPO* myeloperoxidase, *NK *natural killer cells, *NLR* neutrophil-to-lymphocyte ratio, *TNF* tumor necrosis factor, *Tx* thymectomized patients, *vWF* Von Willebrand factor, *y* year of life

**Fig. 2:**
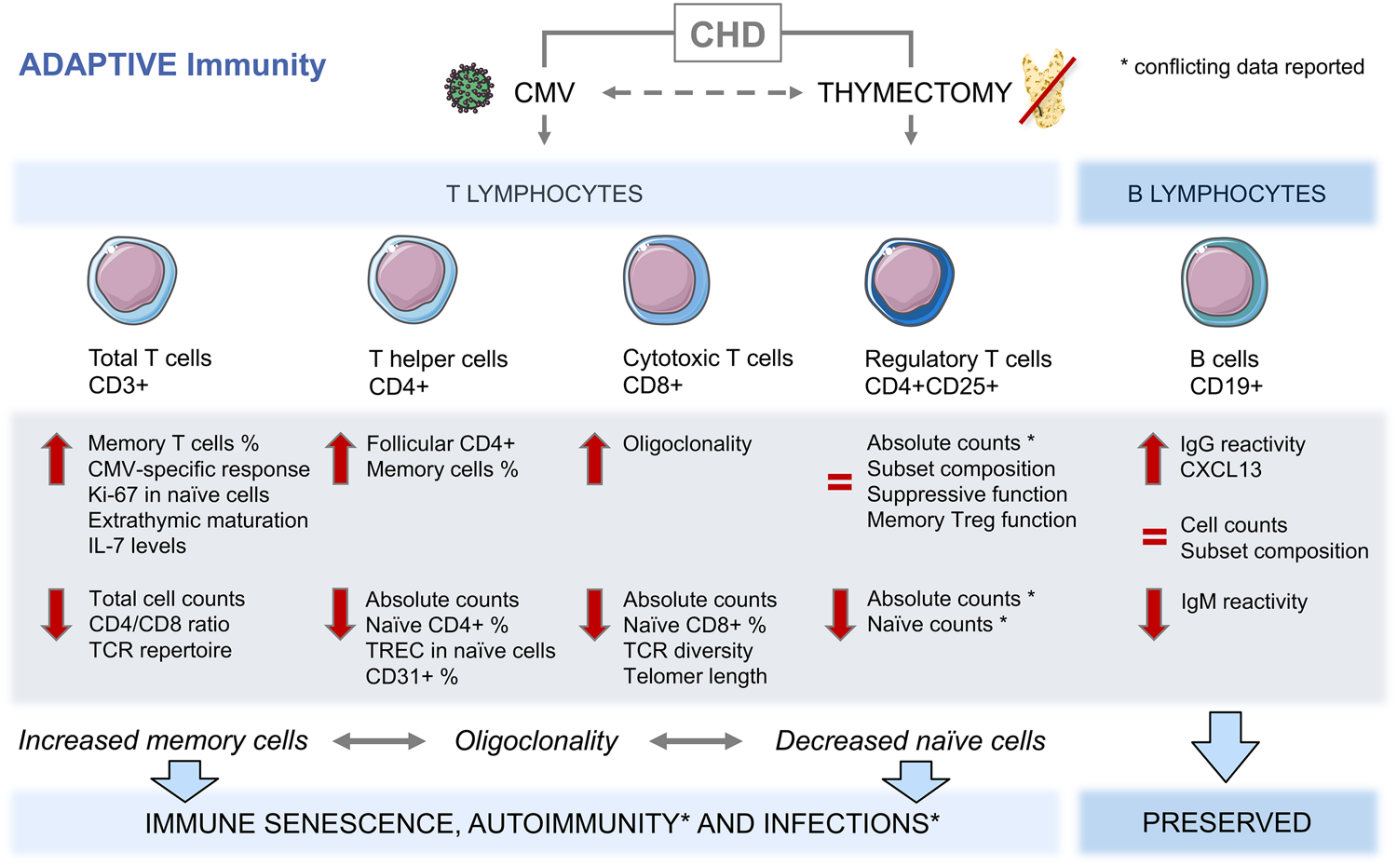
**Adaptive immune mediators.** Congenital heart disease (CHD)-associated early thymectomy and CMV seropositivity are related to alterations of adaptive immune effectors, such as increased memory and decreased naïve T cells and signs of oligoclonality. These changes are contributing to premature immune senescence and increased incidence of autoimmune diseases and other comorbidities. B cell compartments are not affected itself. Red flashes indicate increased or decreased parameters; equal sign indicates unchanged parameters in CHD compared to controls. ^%^Indicates proportional increase of the compartment. *Indicates conflicting data, discussed in the appropriate section of the text. Servier Medical Art PowerPoint templates were used for graphical illustration. *CD* cluster of differentiation, *CHD* congenital heart disease, *CMV* cytomegalovirus, *Ig* immunoglobulin, *IL* interleukin, *TCR* T cell receptor, *TREC* T cell receptor excision circles, *Treg* regulatory T cells

**Fig. 3:**
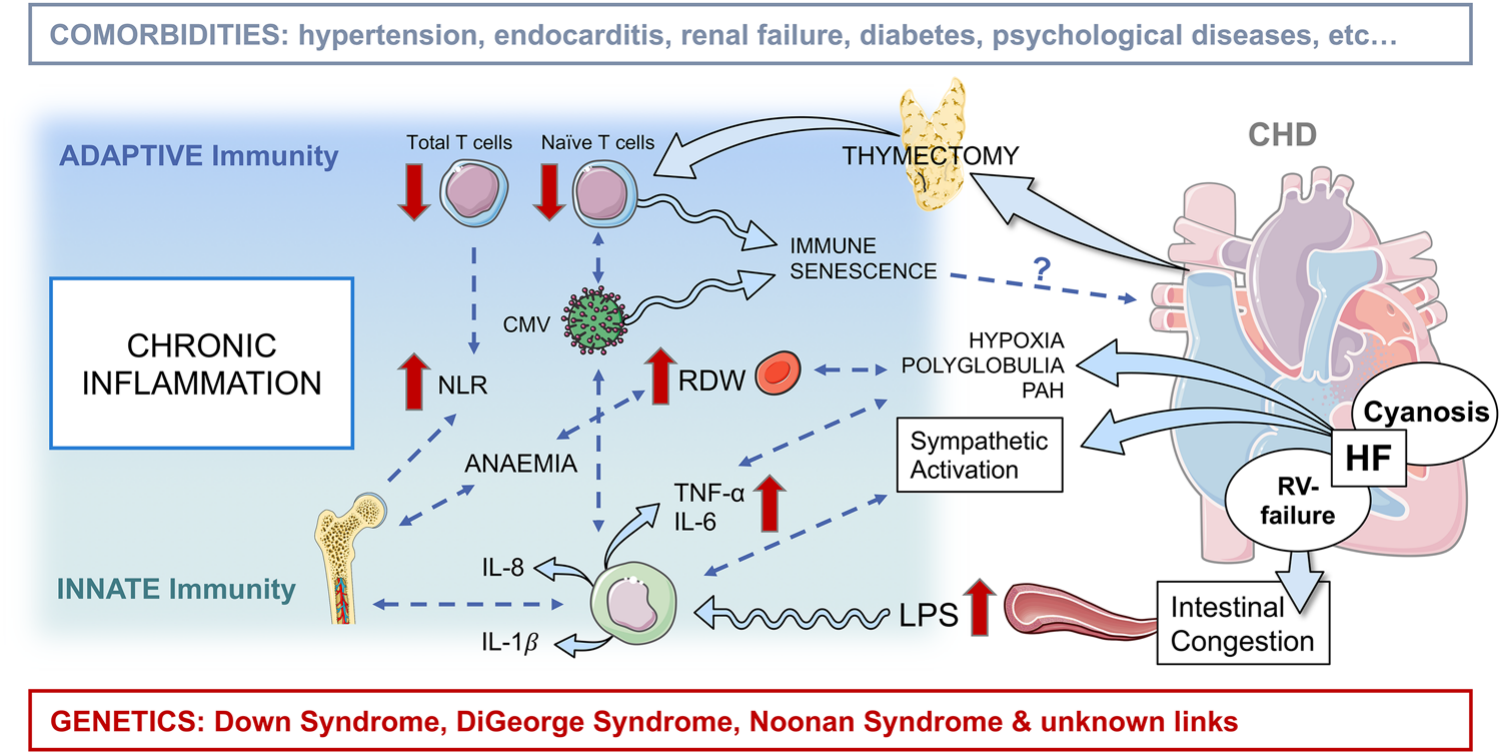
**Central illustration.** Immunity and inflammation represent key players in heart failure and associated comorbidities in CHD. Thymectomy affects T cells of the adaptive immune system. Whereas heart failure-induced congestion, hypoxia and sympathetic activation can influence especially effectors of the innate immune system, such as monocytes, resulting in increased cytokine levels and immune activation. Servier Medical Art PowerPoint templates were used for graphical illustration. *CHD* congenital heart disease, *CMV* cytomegalovirus, *HF* heart failure, *IL* interleukin, *LPS* lipopolysaccharide, *NLR* neutrophil-to-lymphocyte ratio, *PAH* pulmonary hypertension, *RDW* red blood cell distribution width, *RV* right ventricular, *TNF* tumor necrosis factor

Heart failure relates to chronic inflammation mediated primarily by innate immune activation. Indeed, alterations regarding cellular and humoral effectors of the innate immunity were observed in CHD (Tables 1 and 2, Fig. 1). Immune alterations of the adaptive immune system are present in patients with CHD and may result from infant thymectomy (Fig. 2). The detailed changes in innate and adaptive immunity are described below discussing possible causes or contributing factors to CHD-HF, such as congestion, hypoxia, open-heart surgery, genetic syndromes, and comorbidities, as condensed in Fig. 3.

### Heart failure, congestion, and hypoxia as immune stimulators in CHD

The various hemodynamic conditions of severe CHD can activate the immune system by causing myocardial injury, hypoxia, edema, and hypoperfusion, contributing to chronic inflammation and the onset or progression of complicating comorbidities in CHD. The other way around, remote injury, and systemic inflammation can induce a local myocardial inflammatory response mediated by cardiac macrophages [10]. Thus, systemic inflammation and HF are mechanistically linked. A characteristic issue in some CHD is right ventricular dysfunction, which results in congestion as well as in peripheral and intestinal edema. Hypoperfusion and bowel wall edema facilitate bacterial lipopolysaccharide (LPS) translocation from the gut lumen into blood circulation [11–13]. Indeed, several studies found that blood bacterial endotoxin levels (e.g., LPS) related to HF characteristics and post-surgery outcome in CHD patients [13–15]. In adult patients with congenital heart disease (ACHD), elevated pro-inflammatory cytokines and bacterial endotoxins related to HF functional status and peripheral congestion [16]. Through stimulation of toll-like receptor (TLR)-expressing immune cells, such as monocytes, bacterial endotoxins can induce a significant cytokine production and inflammatory immune response, maintaining an inflammatory state. In addition, hypoxia represents a potential immune stimulus in CHD, since it is known that patients with cyanotic heart defects display particularly elevated TNF-α, IL-6, and higher hsCRP [16–18]. Hypoxia itself induces the transcription of several inflammation-promoting genes, such as NF‑κB and TLRs, via the alpha subunit of the hypoxia-inducible transcription factor (HIF-1α) [19]. In cyanotic children with CHD, myocardial induction of the inflammation-related IL-6-JAK-STAT and NF-κB pathways was demonstrated including increased IL-6, C-myc, and suppressor of cytokine signalling-3 (SOCS3) mRNA levels [20]. Hypoxia-induced SOCS3 expression can be stimulated by inflammation and infection and is involved in the myocardial adaptation to chronic hypoxia in CHD [20, 21]. Induction of SOCS3 was reported to be a promising treatment strategy in rodent acute inflammation models [22, 23]. Thus, SOCS3 regulation in hypoxia and inflammation of CHD patients represents an interesting translational therapeutic target. Cyanosis and hypoxia are predominantly the result of some distinct cardiac malformations, such as shunt defects and Fontan circulation, sometimes complicated with pulmonary hypertension, Eisenmenger syndrome, and right heart failure. In infants with CHD, several inflammatory molecules, such as RANTES and macrophage migration inhibitory factor (MIF) related to pulmonary congestion or pulmonary vascular resistance, respectively—mechanisms known to be involved in the pathophysiology of pulmonary hypertension [24]. Therefore, hemodynamics of cardiac malformations and inflammatory pathways seem to impact each other in HF of CHD.

### Thymectomy relating to long-term adaptive immune alterations

The majority of CHD patients underwent cardiac surgery during childhood with an increasing proportion of operated CHD in the last decades [25]. Surgical access for corrective or palliative operations often involves a sternotomy and incidental partial or complete removal of the thymus. However, the amount of eliminated tissue highly depends on the individual anatomy, surgeons, hospital standards, type of surgery, and patient age [26]. Thus, residual thymus size after early surgery varies between 0 and 50% of the initial mass, mainly caused by resting tissue of the upper poles [25, 27]. The thymus has its highest activity pre- and postnatally with a subsequent decrease during a person’s lifespan as the organ physiologically involutes and degrades into fatty tissue [26]. Profound immune deficiency is known to result from complete thymus aplasia during fetal development, as caused by genetic syndromes, e.g., DiGeorge syndrome [9, 28]. This raised the question about the long-term effects of incidental thymectomy due to open heart surgery in CHD. Despite the observed profound abnormalities of the T cell compartment and composition in early incidentally thymectomized CHD (Fig. 2, “17” section), several short-term studies have neither observed any clinically relevant immune deficiency nor elevated incidence of autoimmune diseases in comparison with age-matched controls [8, 29–32]. However, Halnon et al. investigated autoimmune events in CHD and found elevated anti-dsDNA levels and possibly a higher incidence of atopic skin reactions in children after early sternotomy [33]. The majority of the published studies are limited by the small numbers of cases and exhibited wide differences in age at and time since first open-heart surgery, which hinders an interpretation and comparison of the results. Considering the timepoint and the time passed since surgery is crucial in terms of the dynamic balance of T cell homeostatic pathways. An insufficient follow-up time might explain the various short-term studies failing to document a clinical impact of thymectomy in CHD. In contrast, the only large long-term investigation found several clinically relevant consequences of thymus removal. In this retrospective Swedish population-based cohort study, the authors investigated the incidence of autoimmune, infectious and atopic diseases as well as malignancies in CHD in relation to early thymectomy before the age of 5 years. Incidentally thymectomized patients had an increased risk for autoimmune diseases, hypothyroidism, and type 1 diabetes as well as for the occurrence of infections compared to a surgery control group receiving early cardiac surgery without thymectomy. Compared to a sex- and age-matched general population, thymectomized CHD patients more frequently developed cancer, autoimmune diseases, and atopic diseases and had a markedly higher risk for relevant bacterial and viral infections (63.1 vs. 23.1%) [25]. Complex and severe malformations generally require open-heart surgery more often and earlier in life. Hence, HF and thymectomy cannot be regarded strictly separately and might both play an important role in the observed immune alterations and comorbidities in CHD. Whether thymectomy and subsequent T cell alterations do in fact influence HF development or progression remains still unclear. As the discussed Swedish study results have not been confirmed elsewhere, further prospective clinical trials are needed to investigate the long-term consequences of total or partial thymectomy in CHD. Possible implications for the prevention and therapy of T cell deficiency and subsequent comorbidities as well as for the COVID-19 outcome will be discussed below.

### Genetic syndromes relating to CHD, inflammation, and immune alterations

The most common genetic syndromes linked to congenital heart malformations are Down syndrome, DiGeorge syndrome, and Noonan syndrome. These have also been associated with immune deficiencies, senescence, and increased incidence of myelo- and lymphoproliferative malignancies. This raises the question about potential underlying links between cardiac and immune developmental biology. For instance, in coronary or heart valve development embryonic macrophages play a crucial role [34, 35]. However, the specific functions of macrophages or other immune cells in cardiac development and malformations are widely unknown.

#### Down syndrome

Down syndrome (DS, Trisomy 21) is associated with CHD, immune dysfunction, senescence, and precocious aging. About 40–50% of DS patients have congenital heart defects, mostly septal or atrioventricular canal defects [9]. DS comprises intrinsic immune alterations such as abnormalities in thymus size and structure as well as in T and B lymphocyte function [36]. This might be a central underlying factor contributing to the well-known high frequency of airway infections during youth and autoimmune disorders later in life, pointing to an immune dysregulation in DS [36].

#### DiGeorge syndrome

DiGeorge syndrome (22q11DS) is also related to congenital heart malformations and characterized by total or partial thymic aplasia resulting in an immunodeficiency and common clinical manifestations of recurrent infections and autoimmune diseases [37]. CHD can be found in 75–80% of patients with DiGeorge syndrome representing a major cause of mortality [9]. Deficiencies of genes encoded in the deleted 22q11 region, such as TBX1 and CRKL have been found to be responsible for associated cardiovascular, renal, and thymic malformations [38]. Tbx1-deficient mice were characterized by developmental anomalies including thymic hypoplasia and cardiac outflow tract malformations [39]. Genes downstream of the transcription factor TBX1 during pharyngeal development are coding for the chemokine CXCL12 and its receptor CXCR4. Impaired CXCR4 signaling lead to malformations as seen in 22q11DS, suggesting an important role of the CXCL12/CXCR4 axis in its etiology [40].

#### Noonan syndrome

Noonan syndrome is characterized by various types of CHD or hypertrophic cardiomyopathy next to cognitive and growth restrictions as well as skeletal and hematological defects including an increased risk for leukemia [9]. Cytokine hierarchy was assumed to be related to the development of pathological LV hypertrophy as observed in a Raf1 mutant Noonan syndrome mouse model [41]. In this context, postnatal TNF inhibition normalized the existing hypertrophy in vivo suggesting TNF/IL-6 signaling as promising therapeutic targets in 8 caused by kinase-activating RAF1 mutations [41].

### Comorbidities relating to inflammation and immune alterations

Chronic inflammation as well as immune alterations can be the causes and the consequences of comorbidities associated with CHD. Especially during early stages of HF, it has been suggested that inflammation represents a crucial common pathophysiological link between HF and other diseases [42]. A characteristic issue of CHD patients are clinically relevant thromboembolic events and altered hemostasis on the basis of abnormal hemodynamics, comorbidities, polycythaemia, impaired platelet number and function, influenced by inflammation and immune activation [43]. Furthermore, a high prevalence of relevant extra-cardiac comorbidities was noted in ACHD, affecting 95.2% of patients [44]. In particular, the prevalence of various inflammation-related diseases, involving pulmonary, renal, metabolic or neurologic function, was several times higher in ACHD compared to the general population [44, 45]. Directly caused by immunity, an elevated risk for certain autoimmune diseases, infections, cancer, and asthma has been detected in CHD [25]. Comorbidities were strongly associated with higher mortality and hospital admission rates, especially in aging ACHD [44]. In CHD-HF, inflammation could be a possible factor linking CHD-HF to these comorbidities but has not been focused on adequately in clinical routine for diagnostic and treatment purposes.

## Innate immunity in congenital heart disease

The initial immune activation in HF is mainly driven by the innate immune system [42]. Innate immunity can be seen as the first line of defense including physical, chemical, cellular, and humoral barriers and protection strategies. Several insights have been gained regarding humoral and cellular immune activation in CHD (Fig. 1).

### Cytokines and chemokines

The current literature regarding cytokines, chemokines, and parameters of systemic inflammation in CHD is summarized in Table 1 for children with CHD (CCHD) and Table 2 for ACHD. According to these insights, it can be stated that systemic inflammation is present in CHD and is associated with HF characteristics. Some studies illustrated that successful therapy of CHD-HF induced a normalization of inflammatory signaling and a decrease of blood cytokine levels [24, 46, 47]. In human whole heart tissue of CHD patients, an activation of the NF‑κB pathway has been reported at comparable levels to non-congenital HF [48]. Thus, major inflammatory pathways seem to be activated in both entities of HF. However, on the whole, chemokines and their receptor expressions have not yet been extensively investigated in CHD. In children with CHD, elevated serum RANTES (CCL5) and MIF levels have been observed next to decreased angiogenic chemokine GROα concentrations related to pulmonary impairment. Furthermore, elevated eotaxin plasma levels, a CC chemokine subfamily of eosinophil chemotactic proteins, have been revealed in young ACHD [14]. Further research regarding for example CXCR4 or fractalkine and its receptor CX3CR1 might be interesting, as they regulate monocyte recruitment, platelet activation, and inflammation in cardiovascular diseases.

**Table 1:** Circulating markers of inflammation in children with congenital heart disease (CCHD)

Patient cohort: **CHILDREN**	Inflammation-related parameters	Results/associations	Interpretation/limitation	Reference
CCHD undergoing surgical repair, mean age 59 days (*n* = 30)	Plasma endotoxin, LBP, IL-6	40% had **↑** LPS at baseline; LBP and IL-6 ↑ following surgery; **↑** LPS associated with worse outcome	Endotoxemia at baseline and surgery-related, associated with outcome	Lequier et al. [13]
CCHD with ASD, VSD undergoing cardiac catheterization, NYHA I + II (*n* = 27); controls (*n* = 8)	TNF-α in relation to NOx	TNF-α correlated with NOx but not with CI or cardiac output; TNF-α levels ↑ in ASD compared to control	Basal release of NO in shunt lesions **↑**; TNF-α potential activator of iNOS in CCHD	Takaya et al. [88]
Infants with left-to-right shunts and **↑** pulmonary circulation (*n* = 15) vs. cyanotic heart defects with** ↓** pulmonary blood flow (*n* = 11)	sTNF-R1, sTNF-R2	Left-to-right shunts had **↑** sTNF-R levels vs. heart defects with cyanosis; sTNF-R levels **↓** under propranolol β-blockade therapy (for 3 weeks, *n* = 4)	Immune mechanisms and cytokine production involved in HF of CCHD; differences between functional lesions	Buchhorn et al. [47]
CCHD undergoing surgical repair (*n* = 61)	Plasma endotoxin, LBP, IFABP, CRP, monocytic TLR4, TLR2 and HLA-DR	↑ levels of IFABP and endotoxin in duct-dependent defects prior to surgery; ↑ gene expression of TREM-1, IL-10, and TLR signalling in myeloid cells and **↓** monocytic TLR4, TLR2 and HLA-DR post-surgery	Studying acute inflammation due to surgery and cardiopulmonary bypass promoting disruption of gut barrier function and immune stimulation	Pathan et al. [15]
Cyanotic (*n* = 60) vs. acyanotic (*n* = 60) CCHD and healthy control children (*n* = 30)	TNF-α, IL-6, CRP, VEGF, caspase 3	↑ TNF-α, IL-6 and CRP in CCHD and even ↑ in cyanotic patients; caspase 3, VEGF, and troponin T ↑ in cyanotic CCHD	CCHD is inflammation related; cyanosis is potential stimulus for immune activation and tissue damage	Nassef et al. [17]
Repaired CoA (*n* = 10), healthy age-matched controls (*n* = 10), < 12 years	TNF-α, IL-6, IL-10, sICAM-1, sVCAM-1, e-selectin, sFas	↑ TNF-α, IL-6, IL-10 and sFas in CoA patients, unchanged levels of soluble adhesion molecules	Inflammatory activation and apoptotic processes in CoA, despite early cor-recting surgery and normal LV function	Moutafi et al. [89]
CCHD (*n* = 67), a subgroup receiving percutaneous procedure (*n* = 37), healthy controls (*n* = 20)	TNF-α, IL-6, ghrelin	↑ Serum levels of TNF-α, IL-6 and ghrelin compared to controls; these levels** ↓** 3 months after percutaneous ASD, VSD or PDA closure	Interventional treatment of heart defects can **↓** inflammatory activation; limitation ↑ loss of follow-up	Yadav et al. [46]
Cyanotic (*n* = 30) vs. acyanotic (*n* = 30) CCHD, healthy sex- and age-matched controls (*n* = 30)	TNF-α, IL-6, IL-10, IL-12, IL-18	↑ TNF-α, IL-6 and IL-18 vs. controls; in cyanotic CCHD ↑ TNF-α, IL-6 and RVSP; TNF-α and IL-6 related to RVSP; no differences in IL-10 and IL-12	Pro-inflammatory cytokines relate to RV pressure; cyanotic CCHD have ↑ RVSP and hypoxia	Noori et al. [90]
Young CCHD with shunt defects (*n* = 47), 0.2–3.1 years	15 cytokines and chemokines	RANTES ↑ in pulmonary congestion; MIF ↑ related to ↑ vascular resistance; IL-17E ↑ but angiogenic GROα ↓ with age; sildenafil ↑ pulmonary function and ↓ IL-6 and sICAM levels	levels of different inflammatory molecules related to disease severity and pulmonary hypertension, same results in Down Syndrome CCHD	Zorzanelli et al. [24]
Children and adolescents (*n* = 377) divided into cyanotic, acyanotic, and minimal heart defects	hsCRP in relation to BMI	cyanotic CCHD had ↑ hsCRP compared to minimal defects; hsCRP correlated with BMI	Authors suggest fatty tissue as immune stimulus, but did not compare hsCRP in normal vs. obese CHD. Correlation might have confounding comorbidities	Goulart et al. [18]

*ASD* atrial septal defect, *AVSD* atrioventricular septal defect, *BMI* body mass index, *CI* cardiac index, *CoA* coarctation of the aorta, *CRP* C-reactive protein, *GROα* growth-regulated oncogene alpha, *HF* heart failure, *HLA-DR* human leukocyte antigen DR, *IL* interleukin, *iNOS* inducible isoforms of nitric oxide synthase, *IFABP* intestinal fatty acid binding protein, *LPS* lipopolysaccharide, *LBP* LPS-binding protein, *LV* left ventricle, *MIF* macrophage migration inhibitory factor, *NO* nitric oxide, *NOx* nitrate/nitrite, *NYHA* New York Heart Association class, *PDA* patent ductus arteriosus, *RANTES* regulated on activation normal T cell expressed and secreted, *RVSP* right ventricular systolic pressure, *sFas* soluble protein Fas, *sICAM* soluble intercellular adhesion molecule, *sTNF-R* soluble TNF receptor, *sVCAM* soluble vascular adhesion molecule, *TLR* toll-like receptor, *ToF* tetralogy of Fallot, *TNF* tumor necrosis factor, *VEGF* vascular endothelial growth factor, *VSD* ventricular septal defect

**Table 2:** Circulating markers of inflammation in adults with congenital heart disease (ACHD)

Patient cohort: **ADULTS**	Inflammation-related parameters	Results/associations	Interpretation/limitation	Reference
ACHD (*n* = 52), healthy control (*n* = 18)	TNF-α, IL-6, IL-10, sTNF-R1, sTNF-R2, endotoxin, sCD14	↑ TNF-α, IL-6 and endotoxin compared to control; no differences in sTNF-R, IL-10 or sCD14 vs. control; TNF related to HF severity; TNF-α and IL-6 ↑ in cyanotic ACHD; endotoxin ↑ in ACHD with edema	Immune activation in HF of ACHD; inflammatory cytokines and bacterial endotoxin related to functional status and disease characteristics	Sharma et al. [16]
Young ACHD with TGA and infant thymectomy (*n* = 25), age-matched and old controls (*n* = 29)	IL-1β, IL-8, eotaxin	↑ Pro-inflammatory cytokine levels compared to controls, similar levels as in the elderly cohort	Next to T cell immune senescence, pro-inflammatory state in young ACHD	Sauce et al. [14]
Atrial switch operation for transposition of the great arteries (*n* = 27), controls (*n* = 20)	TNF-α, annexin 5	↑ TNF-α and annexin 5 levels compared to control; annexin 5 correlated with TNF and systemic ventricular global longitudinal strain	Impaired myocardial deformation relating to ↑ cytokine levels	Lai et al. [91]
ACHD (*n* = 144) during a mean 4.8-year follow-up	IL-6, blood RDW	↑ RDW correlated with mortality; the high RDW group had ↑ IL-6 levels, ↑ IL-6 in anaemic ACHD	Pro-inflammatory IL-6 may inhibit EPO-induced erythrocyte maturation	Miyamoto et al. [92]
Bicuspid aortic valve patients (*n* = 139)	NLR	NLR correlated with ↑ ascending aorta diameter and **↓** LVEDD, LVESD, and LVEF in patients with dilated aorta > 3.9 cm (*n* = 21), but not in < 3.9 cm group	Limitations: small > 3.9 cm group size and no mean NLR difference between < 3.9 vs. > 3.9 cm	Kasapkara et al. [93]
ACHD (*n* = 103) divided into 1- or 2- ventricle physiology and into systemic left or right ventricle	hsCRP, IL-6, hsTNF, sTNF-R1, sTNF-R2, NOR	↑ IL-6, sTNF-R1 and NOR related to NYHA class and were independent predictors of mortality in ACHD; distinct profiles in the morphologic groups	Differences in inflammation and neuro-hormonal activation between heart physiology groups predicting outcome	Miyamoto et al. [94]
Teenager and ACHD undergoing transcatheter shunt closure (*n* = 200); 13–41 years; controls (*n* = 200)	TNF-α, IL-6, IL-8, IL-10, JNK, and NF-κB signalling pathways	**↓** IL-10 and ↑ TNF-α, IL-6, IL-8 in CHD prior to and 6 months-, but **↓** to control level 1y after closure; ↑ JNK and NF-κB activation in CHD; JNK + NF-κB inhibitor treatment restored cytokine levels in pig CHD model	JNK and NF-κB signaling pathways were activated in CHD, transcatheter closure treatment may**↓** the activation	Fan et al. [80]
ACHD outpatients (*n* = 602)	RDW	↑ RDW was associated with cardiovascular events, independently of age, sex, CRP, and NT-proBNP	Prognostic relevance of RDW in large ACHD cohort	Baggen et al. [7]
ACHD outpatients (*n* = 707)	hsCRP	↑ hsCRP was associated with adverse outcomes; 25% of ACHD had hsCRP > 3 mg/L	Systemic inflammation present in a quarter of ACHD outpatients relating to all-cause mortality and CV events	Opotowsky et al. [6]
ACHD outpatients (*n* = 590), median follow-up 5.8 years, 90% NYHA I	sST2	sST2 related to the composite endpoints, associations rather in complex lesions and women	Only in 10% of study patients ↑ sST2, to healthy cohort for outcome analysis	Geenen et al. [79]
ACHD outpatients (*n* = 209)	Monocyte subsets, CRP, NLR, NOR	↑ Mon2 CD16 + monocytes related to NYHA class and ↑ RVSP, hypoxia related to RVSP, ↑ NLR, ↑↑ Mon2 in defects with severe congestion/ cyanosis	congestion and hypoxia as possible immune stimuli in ACHD with ↑ RVSP	Wienecke et al. [54]
ACHD outpatients (*n* = 30), age gender and BMI-matched controls (*n* = 30)	TNF-α, IL-6	IL-6 correlated with 6MWD, dyspnea, biomarkers of heart, lung, and inspiratory muscle function	Chronic systemic inflammation is associated with exercise intolerance caused by heart and lung dysfunction	Spiesshoefer et al. [95]

*BMI* body mass index, *CV* cardiovascular, *EPO* erythropoietin, *HF* heart failure, *hsCRP* high-sensitivity C-reactive protein, *IL* interleukin, *JNK* c-Jun N-terminal kinase, *LVEDD* left ventricular end-diastolic dimension, *LVESD* left ventricular end-systolic dimension, *LVEF* left ventricular ejection fraction, *Mon2* CD14+ +CD16+ monocyte subset, *NF-κB* nuclear factor kappa B, *NLR* neutrophil-to-lymphocyte ratio, *NOR* noradrenaline, *NT-proBNP* N-terminal prohormone of brain natriuretic peptide, *NYHA* New York Heart Association class, *RDW* red cell distribution width, *RVSP* right ventricular systolic pressure, *sST2* soluble suppression of tumourigenicity-2, *sTNF-R* soluble TNF receptor, *sCD14* soluble CD14, *TNF* tumor necrosis factor, *6MWD* 6-min walking distance

### Neutrophils

Neutrophils are produced and released by the bone marrow and represent the most abundant type of blood immune cells during the early innate immune response, acting towards host defense by degranulation, phagocytosis, and cell recruitment to the inflammation site. Higher blood neutrophil counts have been observed in CHD [29]. In addition, patients with cyanotic heart disease exhibited increased platelet activation next to elevated neutrophil elastase levels, a marker for neutrophil degranulation reflecting cell activation [49]. Several factors associated with hypoxia and HF might increase neutrophil activation, such as endothelial dysfunction, platelet activation, and inflammatory cytokines [49]. In terms of neutrophil function, no significant difference was found, indicating an intact neutrophilic innate immunity in CHD patients [50].

### Monocytes/macrophages

Monocytes/macrophages represent powerful mediators of inflammation and innate immunity involved in cytokine production and cell recruitment. In HF and myocardial infarction, monocytes/macrophages play key roles in healing and remodeling, exhibiting both beneficial and detrimental functions [5, 51, 52]. This might be explained by the time course of activity and the existence of different monocyte and macrophage subtypes. In detail, three human monocyte subgroups have been described, CD14^++^CD16^−^ (Mon1, classical), CD14^++^CD16^+^ (Mon2, intermediate), and CD14^+^CD16^++^ (Mon3, non-classical) monocytes, showing different phenotypic and functional characteristics [53]. We found Mon2 and Mon3, seen as rather proinflammatory monocytes, to be elevated and increasing with HF grade in ACHD outpatients. Highest Mon2 levels were observed in patients with native single ventricle, Eisenmenger syndrome and pulmonary hypertension—defects associated with severe congestion and cyanosis [54]. Further functional studies concentrating on monocytes and their role in CHD-HF and risk stratification are still lacking.

### Natural killer cells

Classified as innate lymphoid cells, natural killer (NK) cells can act immediately without specific antigen recognition to eliminate virus-infected or malignant cells. Hence, this cell lineage is essential for the body’s anti-tumor strategies. NK cells are bone-marrow-derived and independent of T cell and thymus development as they do not undergo thymic maturation [55]. Accordingly, a number of studies did not find any differences in CHD NK cell number or composition, neither shortly nor years after thymectomy [14, 31, 56]. In contrast, in 1996, Ramos et al. reported an increased number of the CD16^−^CD56^bright^ NK subset and a general increase of NK activity, but only in children who had been thymectomized during the first year of life. The authors suggest that the absence of the thymus promoted the differentiation of the common T and NK cell precursors into NK cells [57]. These conflicting data will need further investigation.

### Mast cells

Mast cells are bone marrow-derived and play an important role in allergic reactions and parasite defense. An increased expression of mast cell chymase in the lung tissue of patients with CHD was reported to be associated with early pulmonary vascular disease [58]. Nevertheless, the impact of mast cell activation and its pathways in CHD and associated pulmonary hypertension are still unclear.

### Platelets

Activated platelets are known to secrete high amounts of cytokines and chemokines, which further promote cell activation and the subsequent recruitment of other immune cells [59]. Increased platelet activation was found to correlate inversely with arterial oxygen saturation of cyanotic patients and with a lower baseline surface density of the platelet adhesion receptor glycoprotein Ib (CD42) in children with cyanotic heart disease [60]. Other studies described higher platelet microparticle and P-selectin expression as well as decreased percentage of ADP-induced platelet aggregation correlating inversely with hematocrit in cyanotic children with CHD [43, 61]. Von Willebrand factor antigen, considered as a marker of endothelial dysfunction, was shown to be significantly elevated in cyanotic compared to acyanotic CHD [43], suggesting that hypoxia, polycythemia, shear stress, and endothelial dysfunction trigger platelet activation in cyanotic CHD. However, hypoxia-independent mechanisms could also play a role, as in acyanotic patients increased platelet activation was found, too. In a large study of ACHD patients mean platelet volume (MPV) did not correlate with major adverse events, although patients had significantly lower platelet counts compared to controls [62]. The mechanisms behind reduced platelet counts have not been investigated, and the clinical implication remains unclear. Hemodynamics, increased platelet consumption and chronic inflammation might contribute to this observation.

## Adaptive immunity in congenital heart disease

Central effectors of the adaptive immune defense are lymphocytes, including B and T cells. T cells are initially produced in the bone marrow, but maturation into self-tolerant naïve T cells occurs in the thymus, comprising important screening steps to avoid autoimmunity. Simplified, T cells can be divided into CD4^+^ T helper cells and CD8^+^ cytotoxic T cells. T helper cells support other pathogen-eliminating immune cells, while the main function of cytotoxic T cells is the identification and elimination of virus-infected or malignantly degenerated cells by the induction of apoptosis. To improve and accelerate the adaptive immune reaction upon reinfection, both B and T cells can produce memory cells. However, in the context of new, unknown pathogens, antigen-presenting cells need naïve T cells who can be primed. Therefore, the number of memory cells increases, and the number of naïve cells decreases during a person’s lifespan due to pathogen contact and thymic involution [63, 64].

Adaptive (auto)immunity plays a crucial role in cardiac remodeling and heart failure progression. In various cardiovascular diseases, an activation, reactivation, or even persistence of the inflammatory cascade by the adaptive immune system has been shown including T cell activation, tissue infiltration, and expansion. Especially, the interplay between both adaptive and innate immune mediators gained much interest in cardiovascular research and bears broad therapeutic potential [63]. For instance, CD4^+^ T cells are involved in disease progression toward heart failure in murine models of hypertrophy or myocardial infarction [63, 65, 66]. T cell activation is suggested to occur in reaction to self-antigen presentation. In fact, recent insights show that myocardial injury is associated with anti-heart autoantibodies and characteristics of ongoing autoimmune responses [66, 67]. Activated CD4^+^ T cells are known to further differentiate into distinct effector T cell subsets (T_H_1, T_H_2, T_H_17, T_reg_, etc.) having specific properties and functions during acute or chronic myocardial inflammation, e.g., cytokine production, B cell activation, immunomodulation or endothelial cell activation [63]. The role of effector T cells in HF of CHD and the impact of thymectomy on auto-immune related processes upon myocardial injury represent emerging research topics, which have not been addressed scientifically, possibly due to lack of appropriate animal models. Described changes in adaptive immune mediators of incidentally thymectomized CHD patients are illustrated in Fig. 2.

### T helper and cytotoxic T cell compartments

The thymus is the main source of T cells during youth and remains responsible for a low-level renewal of T cells up to the 6th decade of life. Hence, thymectomy especially during early stages of life can impair T cell production profoundly. Several studies revealed different abnormalities of the adaptive immune system in CHD patients who underwent thymectomy by open-heart surgery, already summarized by Roosen et al. [8]. We will add some new insights and highlight the main T cell variations related to infant sternotomy. In early thymectomized CHD, reduced total and especially naïve CD4^+^ T helper and CD8^+^ cytotoxic T cell compartments were reported, which persisted at least 2 decades after initial surgery [14, 31, 56]. Furthermore, these patients have a shorter T cell but unchanged B cell telomere length compared to controls and showed a diminished T cell receptor repertoire with signs of oligoclonality as well as augmented memory T cell levels [32, 56]. These immune alterations are described as immune senescence and represent the reason for adaptive immune deficiency in the elderly general population [68]. Thymic tissue regeneration post-surgery might occur to a certain degree in some patients [69]. However, the alterations of immune cell composition can be directly linked to the amount of thymic tissue removed as patients with complete thymectomy had significantly lower or undetectable T cell recombination excision circles (TREC) levels, a marker for the extent of thymopoiesis, compared to those with residual thymus tissue [56, 70]. In thymectomized CHD, lower TREC levels of naïve T cells and less recent thymic emigrants (CD31^+^) in the helper T cell subset indicate a shift toward peripheral clonal expansion in order to compensate the lacking thymic output [14, 56, 69, 71, 72]. In addition, another study underlining the changes seen in T cell homeostasis described elevated plasma IL-7 levels and a negative correlation between absolute CD4^+^ T cells and IL-7 [31]. IL-7 is known to enhance thymopoiesis and to regulate the naïve T cell compartment. However, the maintenance of IL-7-induced homeostatic proliferation of naïve T cells requires a persistent thymic activity. The absence of thymic tissue might therefore impact T cell reconstitution and diversity throughout life [55, 73]. Loss of naïve T cells and consecutive accumulation of oligoclonal memory cells was also directly associated with cytomegalovirus (CMV) infections in thymectomized ACHD [14]. CMV infections can affect adaptive and innate immune mediators and play an important role in mechanisms of immune senescence. CMV is associated with increased mortality in the elderly and has been described as a promoter of cardiac diseases [74, 75]. CMV seroprevalence and its impact in CHD are unknown but deserve further research.

### Regulatory T cells

Regulatory T cells (Treg) have increasingly gained attention as they are potent regulators of the immune system and exhibit strong immunosuppressive functions. Like other T cells, Tregs are released from the thymus as a naïve phenotype and generate a mature suppressor memory Treg compartment throughout life. Several groups measuring Treg subsets in thymectomized CHD patients reported conflicting data. Some investigations describe a preserved and unchanged Treg compartment [14, 33, 72, 76]. In contrast, during the first years after thymus removal, diminished total Treg counts were observed next to an increased proportion of activated and cytokine secreting Treg cells [77]. Eighteen years post-surgery, lower total and naïve Treg counts have been measured, but proportions of these cells as well as the Treg subset with highest suppressive potential (CD4^+^CD45RA^-^CD25^+ +^) were unchanged [56]. Further conflicting data exist, regarding some reports about reduced naïve Tregs whereas others found unchanged counts but increased cell turnover [33, 56, 76, 77]. Differences are possibly due to varying patient selection, HF grade, residual thymus tissue, age at and time since surgery. Regarding Treg function, unchanged suppressive activity and expression levels of FoxP3, CTLA-4, and CD39, markers for the suppressive function phenotype, have been described within memory Tregs [32, 69]. Of interest, the cytokine IL-7, elevated in thymectomized CHD plasma, is involved in the thymus-independent long-term maintenance and function of naïve Tregs [76], suggesting that IL-7-dependent homeostasis can sustain naïve Treg but not naïve T cell compartments. In conclusion, Tregs remain less affected than other T cells and might have normal, unchanged immunosuppressive functions. 

### B cell compartments and function

B cells need T cells for their complete functional capacity; however, maturation is independent of T cells or thymic tissue. Accordingly, the B cell counts, percentages and subset compositions were seen as unaffected in early thymectomized CHD by several reports [29, 31, 77]. Regarding B cell function, van den Broek and colleagues revealed decreased IgM autoantibodies and an increased IgG reactivity toward cardiolipin and leptin, suggesting a deranged self-tolerance. Follicular T helper cell counts as well as the production of the B lymphocyte chemoattractant (CXCL13) were increased. Furthermore, they reported a higher proportion of patients with positive antinuclear antibody (ANA) detection (58% vs. 33% in healthy control). However, the authors did not observe an augmented onset of autoimmune diseases during the first decades of life but consider thymectomized CHD as a population at risk.

## Clinical implications of inflammation and immune alterations in CHD

### Monitoring inflammation in clinical routine

In several cardiovascular and non-cardiovascular diseases, biomarkers of inflammation exhibited prognostic relevance, due to the fact that inflammation and immune regulation are involved in respective disease mechanisms [78]. Accumulating evidence indicates an inflammatory state in some CHD. Recently, high-sensitivity C-reactive protein (hsCRP) was described as an independent predictor for cardiovascular events and all-cause mortality in ACHD. A quarter of all ACHD patients had elevated hsCRP, which was associated with worse functional capacity and markers of heart failure (HF) [6]. In addition, the neutrophil-to-lymphocyte ratio (NLR), suppression of tumorigenicity 2 (sST2), and red cell distribution width (RDW) were related to HF severity or outcome [7, 79]. Presumably, it may be helpful for clinical decision making to identify patients with relevant immune activation in the context of CHD-HF by assessing hsCRP and complete blood counts. Both can be measured easily in clinical or outpatient settings and represent cheap parameters in order to monitor inflammation and to add information for risk stratification. HF therapy success might also be reflected by changes in inflammatory markers. Interventional or drug treatments of HF lead to declining inflammatory cytokine levels in children with CHD and ACHD [46, 47, 80]. According to recent scientific findings, adaptive immune activation including circulating anti-heart autoantibodies might enable to monitor the immune response upon myocardial injury [66, 81]. These autoantibodies represent an interesting potential biomarker in HF of CHD for detecting persistent, dysregulated adaptive immune activation [82]. Further research about underlying pathomechanisms and an evaluation of inflammation-guided HF therapy should be performed.

### Prevention and treatment of T cell deficiency

Potential clinically relevant long-term consequences of early median sternotomy regarding T cell homeostasis have not been investigated sufficiently and need further research. In addition, the impact of the described immune alterations on cardiac remodeling and HF progression in CHD needs to be investigated. The degree of adaptive immune impairment in respective patients could be quantified by determining T cell compartments, markers of immune senescence or measuring TREC levels. To avoid a possible severe impairment of adaptive immunity, surgeons could attempt to preserve as much thymic tissue in situ as possible during open-heart surgery. Strategies evaluating a preparation of the operation field without explantation of the thymus or if necessary replantation of initially mobilized thymus tissue at the end of open-heart surgery might be of clinical interest. To protect physiological T cell production, primary percutaneous or minimally invasive procedures and lateral thoracotomy might be favored if possible. In patients with clinically relevant T cell deficiency, a future treatment option could target the inhibition of immune senescence and the induction of naïve T cell pool regeneration.

### Personalized immunomodulatory therapy

CHD regroups several types and causes of cardiac defects, comorbidities, as well as different therapeutic interventions with or without thymectomy. As every CHD patient presents a very individual disease phenotype, the inflammatory immune profile and alterations might differ to a similar or even wider extent. Personalized immunomodulatory therapy could create a novel treatment option and strategy in CHD-HF. Immunomodulation is a therapeutic approach that recently entered clinical cardiology (CANTOS-trial) but was already used for years in the treatment of autoimmune and inflammatory diseases. Characterizing patients with significant inflammatory activation or immune deficiencies and identifying particular anti-inflammatory or “anti-immune senescence” therapeutic targets for different CHD immune-phenotypes might be a promising step towards a ground-breaking treatment strategy in CHD-HF to improve morbidity and mortality.

### Implications regarding severe COVID-19

Immune changes and inflammation need to be kept in mind regarding the COVID-19 pandemic but have not been considered in recent reports [83]. T cell deficiency and immune senescence could potentially impair the organism’s defense strategies and inflammatory response regulation. Indeed, T cell senescence and chronic inflammation have been considered a major cause for the well-known worse outcome after SARS-CoV2 infection in the elderly [84, 85]. Thus, CHD could be seen as a population at particular risk. However, it remains totally unclear whether the diminished naïve T cell pool in early thymectomized CHD patients might increase their susceptibility to SARS-CoV-2 infections or even lead to a poorer response to respective vaccines [86]. Linked to CHD and immune senescence, Down syndrome patients seem to develop a more severe form of the disease and have a worse outcome upon COVID-19 [87]. In order to investigate risk and outcome, the International Society for Adult Congenital Heart Disease built up a database of ACHD patients who have tested positive for SARS-CoV-2. When considering this, T cell alterations and immune senescence, caused by early open-heart surgery, genetic syndromes or HF, should be taken into account to allow interpretation of future study results.

## Conclusions

Congenital heart disease displays several features of a chronic inflammatory disease and often involves significant immune activation and dysfunction of some lymphocyte subsets that should be considered, especially in the context of the current COVID-19 pandemic and the onset of extra-cardiac comorbidities. It is crucial that cardiologists, pediatric cardiologists and cardiothoracic surgeons are aware of the described immune changes and their potential implications. The still practiced surgery-related removal of thymic tissue should be questioned and further long-term investigations regarding the consequences of thymectomy initiated.

## Data Availability

Not applicable.
